# Toward amino acid typing for proteins in FFLUX

**DOI:** 10.1002/jcc.24686

**Published:** 2016-12-19

**Authors:** Timothy L. Fletcher, Paul L. A. Popelier

**Affiliations:** ^1^ Manchester Institute of Biotechnology (MIB), 131 Princess Street Manchester M1 7DN United Kingdom; ^2^ School of Chemistry University of Manchester, Oxford Road Manchester M13 9PL United Kingdom

**Keywords:** QTAIM, peptides, kriging, force field design, machine learning, quantum chemical topology, atomic charge

## Abstract

Continuing the development of the FFLUX, a multipolar polarizable force field driven by machine learning, we present a modern approach to atom‐typing and building transferable models for predicting atomic properties in proteins. Amino acid atomic charges in a peptide chain respond to the substitution of a neighboring residue and this response can be categorized in a manner similar to atom‐typing. Using a machine learning method called kriging, we are able to build predictive models for an atom that is defined, not only by its local environment, but also by its neighboring residues, for a minimal additional computational cost. We found that prediction errors were up to 11 times lower when using a model specific to the correct group of neighboring residues, with a mean prediction of ∼0.0015 au. This finding suggests that atoms in a force field should be defined by more than just their immediate atomic neighbors. When comparing an atom in a single alanine to an analogous atom in a deca‐alanine helix, the mean difference in charge is 0.026 au. Meanwhile, the same difference between a trialanine and a deca‐alanine helix is only 0.012 au. When compared to deca‐alanine models, the transferable models are up to 20 times faster to train, and require significantly less *ab initio* calculation, providing a practical route to modeling large biological systems. © 2016 The Authors. Journal of Computational Chemistry Published by Wiley Periodicals, Inc.

## Introduction

The 20 natural amino acids can be mixed and matched to create proteins performing all manner of biological function. In essence, peptide chains should be relatively simple to model given their repeating backbone and tendency to form predictable secondary structures. However, in reality, a protein becomes a web of interatomic interactions, many contributing to secondary and tertiary structures that determine protein function. Force fields constructed from harmonic potentials, properly parameterized, have been a popular approach to tackle this degree of complexity. For each type of interatomic interaction, a new potential can be constructed but one that may not be applicable outside of the data used to parameterize it. Meanwhile, (re)parameterization is a costly process that does not guarantee the improvement of a force field's predictive power.

In practice, difficulties in modeling peptides can begin at a more fundamental stage. A single atom is commonly atom‐typed by considering its immediate atomic neighbors, ignoring neighboring (amino acid) residues. However, a given atom does experience significant charge transfer and polarization effects from the neighboring residue, neither of which is well‐handled by traditional force fields. It is known that neighboring residues^1^ have a significant effect on chemical shift, which is commonly related to atomic charge.^2^, ^3^ For example, neighboring amino acids in catalytic triads are often ignored but, surprisingly, these residues are strongly conserved in Nature and are important to the function and structure of the active site.^4^ Surprisingly little effort has been made to show the effects of neighboring residues on the atomic charges used so confidently by modern force fields.

The above concerns are likely to be important to future developments of popular force fields. For example, CHARMM's^5^ atom‐typing process has become increasingly sophisticated but is still focused solely on neighboring atoms with little awareness of nearby residues.^6^ Recent improvements to CHARMM's handling of peptides^7^ and peptoids^8^ have involved reparameterizations of the backbone and torsional parameterizations of the sidechain and amide group. AMBER's^9^ ubiquitous “GAFF” parameter set has demonstrated success but also tends to suffer from a lack of polarization,^10^, ^11^ charge transfer and poor electrostatic interaction energies^12^ such as hydrogen bonding, which is in part due to parameterized charges lacking anisotropy.^13^ GAFF defines a given atom's atom‐type by its element and its immediate, bonded neighbor atoms. Thus, an atom‐type is a specific atom in a small molecular fragment. In both AMBER and CHARMM, more accurate atomic charges can likely be gained through consideration of neighboring resides.

Of course, these oversights linger on due to the great challenges posed by overcoming them. Earlier work in our group^14^ studied the transferability of the intra‐atomic energy of topological atoms in sequences of five amino acids (pentapeptides), and three amino acids (tripeptides), with that of atoms in a single amino acid. This work was carried out for seven peptides built from the same amino acid, and concluded that intra‐atomic energies in tripeptides are representative of those in pentapeptides. Hence, it is sufficient to use tripeptides for constructing atom‐types that can be safely transferred to larger systems such as proteins themselves.

Even just considering an amino acid's immediate neighbors, there are 8000(=20 × 20 × 20) possible natural tripeptide combinations, making handling each of these combinations explicitly quite impractical. While some tripeptide combinations are common, it may be true that others seldom occur, which would guide atom‐typing. Unfortunately, due to the meagre amount of data available, it is difficult to draw meaningful insight into the 8000 possible tripeptides and, more difficult still, to group the tripeptides in a meaningful way. Indeed, even when considering single amino acids, there is disagreement as to their relative frequencies in proteins^15^, ^16^, ^17^, ^18^, ^19^ (Table S1 of the Supporting Information). It is our hope that neighboring amino acids can be grouped in terms of their influence on a central residue and that atom‐types will emerge from these groupings. Amino acids are commonly discussed as being functionally related to one another, such as neutral amino acids, charged amino acids, or aromatic amino acids.^20^ Using chemical intuition to group amino acids is useful for an isolated amino acid but is not so simple when considering how these same amino acids might affect a neighboring residue and how atom‐typing might reflect that effect. Thus, atom‐typing and parameterization is often accomplished by considering experimental data (often crystallographic) but these are not always available or even reliable.^21^ Therefore, we suggest discerning amino acid groupings based on *ab initio* calculation of these neighboring effects rather than relying on chemical intuition alone.

It is with these considerations in mind that we continue the development of FFLUX,^22^ which is a multipolar force field that applies machine learning to properties derived from quantum chemical topology^23^, ^24^ (QCT). The latter approach originates from the “Quantum Theory of Atoms in Molecules” (QTAIM).^25^, ^26^ Changes in molecular configuration cause changes in the electron density, which in turn affect atom‐centred multipole moments. These higher multipole moments (i.e., everything beyond the monopole moment or point charge) capture the anisotropy of the electron density and hence the correct polarization of the system.^27^, ^28^ In the past we have shown FFLUX to be applicable to water clusters,^29^, ^30^ methanol,^31^ *N*‐methylacetamide^32^ and all amino acids^33^, ^34^ including aromatic amino acids,^35^ alanine helices,^36^ and carbohydrates.^37^ In previous publications, we have explored the concept of transferability using the machine learning method kriging^38^ for small molecules and now turn our attention toward the challenge of predicting interatomic electrostatic interaction in proteins. In such systems, polarization has proven to be an important factor in hydrogen bonding^39^, ^40^, ^41^, ^42^, ^43^ and structural determination,^44^, ^45^, ^46^ and many groups work to incorporate these effects into modern force fields.

**Figure 1:**
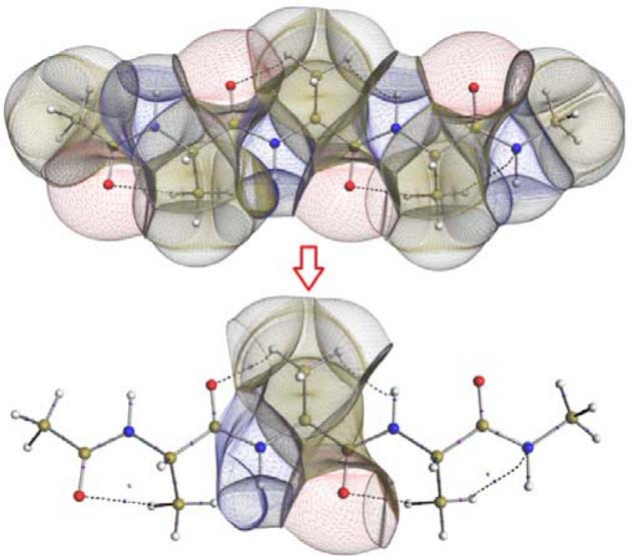
Superposition of the molecular graph and topological atoms in (top panel) “trialanine” (AAA, 42 atoms) and (bottom panel) the central fragment (10 atoms). The space‐filling nature of the atoms makes it easy to isolate molecular fragments. The hydrogen atom that is bonded to the C_α_ is hidden in both panels. [Color figure can be viewed at wileyonlinelibrary.com]

Using QCT, we can partition the electrostatic (and non‐electrostatic) energy of a chemical system into (both physically and chemically) meaningful contributions, without invoking a reference electron density, parameters or molecular orbitals explicitly. QCT defines (topological) atoms with well‐defined boundaries as shown for trialanine (AAA) in Figure 1. The so‐called interatomic surfaces separate the atoms, without overlap or gaps, that is, in a space‐filling manner. All space within the atomic boundary is said to “belong” to the atom and so does the electron density. Atomic properties are then obtained by integration over each atomic volume. These atomic properties are highly sensitive to the extended chemical environment, making them potentially difficult to generalize to other systems. However, it has been shown in our past publications that these atomic charges can be related to molecular geometry using the machine learning method kriging.

It is important to put the QTAIM (and hence QCT) atomic charge briefly in context. We have repeatedly made clear that the QCT atomic charge has not been designed^47^, ^48^ to reproduce an atomic electrostatic potential. Instead, we have always seen charge as the first term of a multipolar expansion of the electrostatic potential or interaction. Many years ago, we have proven^49^, ^50^ that with a sufficient number of terms one can *exactly* reproduce an atomic electrostatic potential. Nevertheless, QTAIM charges are still sometimes criticized for failing to reproduce the electrostatic potential by themselves, although at long‐range even charges alone suffice to obtain the exact electrostatic interaction.^51^ QTAIM charges have also been disparaged for being too large but these criticism has been rebutted.^52^

In a recent publication,^36^ we showed that kriging models for an atom within an alanine unit in deca‐alanine can be generalized to predict properties on any atom within the helix. By taking a fragment of a molecule (such as that in the bottom panel of Fig. 1), a kriging model can be made that predicts charges for an atom in that fragment. If this given fragment is common to many molecules, such as an amino acid in a protein, then the model can be reused in a very large number of chemical systems. For example, the tripeptide Ala‐Ala‐Ala (termed “AAA”) can have its central alanine fragment (Fig. 1, bottom) isolated and models created for it. In theory, these models could then predict alanine charges in the tripeptide Val‐Ala‐Val (VAV). This generalization is the basis of transferability within FFLUX and should allow for large systems to be modeled. Unfortunately, an atom's properties change significantly as its extended environment changes. Hence, an alanine neighboring a tryptophan might differ significantly from one neighboring a valine, thus requiring a separate kriging model. However, models can conceivably be shared if multiple amino acids are proven to have similar influence as neighbors.

In this work, we use FFLUX to investigate three questions important to transferability in FFLUX and force fields at large:

1. Can the natural amino acids be grouped according to their influence on a neighboring residue?
2. Can the influences of neighboring amino acids be accounted for using kriging machine learning?
3. What is the minimum size of a peptide that can be used to train kriging models for predicting atomic charges in a polypeptide chain?

By answering these questions, we can define a methodology for transferability that is sensitive to both local and extended chemical environments while maintaining the simplicity of the standard understanding of atom‐typing in proteins.

The current work involves the *ab initio* calculations of more than 22,000 peptide chains and their QTAIM properties, and builds on past work to develop the FFLUX force field.

## Methods

### Definition of the datasets

**Figure 2:**
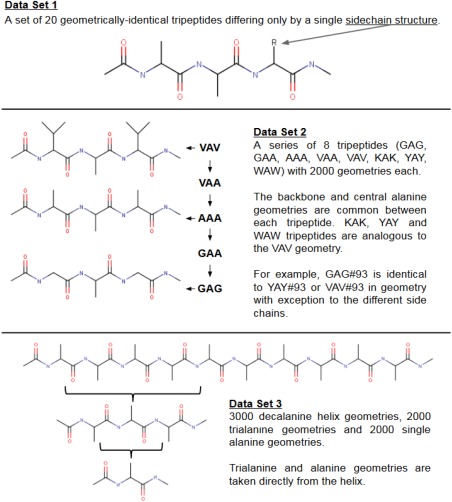
Two‐dimensional illustrations of each the three types of peptide data set. Schematic images of molecules are not representative of their actual geometries. Carbon atoms (black) are not labeled and hydrogen atoms are not shown. [Color figure can be viewed at wileyonlinelibrary.com]

Three data sets are described here, each with a specific purpose. The data sets are illustrated in Figure 2 and outlined below, followed by general computational details.

#### Data Set 1

A set of tripeptides is created and “cleaned” in three dimensions using *ChemSketch*, setting all bond lengths and angles to reference equilibrium values. Each tripeptide consists of a chain of two alanines and one additional amino acid, as shown in Data Set 1 in Figure 2. Thus, every tripeptide in Data Set 1 differs from the others only by the sidechain of its third residue. This data set will show how different amino acid residues (i.e. the third residue) affect the properties of atoms in the central, neighboring residue.

#### Data Set 2

Another tripeptide, VAV (valine‐alanine‐valine), is created and geometry‐optimized by the program GAUSSIAN09.^53^ The resulting energy minimum is used as a template from which hundreds of distorted geometries are generated through the molecule's normal modes of vibration based on in‐house methodology.^37^, ^54^ No bond distance or valence angle is distorted beyond ±15% of its original value in the minimum energy geometry. This tripeptide (VAV) can then be converted to other tripeptides by substituting relevant sidechains to create the tripeptides VAA, AAA, GAA, and GAG. Furthermore, three tripeptides, KAK, YAY, and WAW (Lysine (K), Tyrosine (Y), and Tryptophan (W)) are created in the same manner. Data Set 2 will help determine whether a kriging model can predict the properties of a central alanine when the neighboring residues are (or are not) part of the same group of amino acids as those in the training set.

#### Data Set 3

A final set of molecular geometries are based on a 3_10_ alanine helix taken from the Brookhaven Protein Data Bank (PDB) (entry IL36.pdb). Hydrogens were appended to the helix in the appropriate places using the program GAUSSVIEW with default bond angles and lengths before geometry‐optimizing it using GAUSSIAN09 at HF/6‐31 + G(d,p) level. A single alanine unit can be cut out of the helix and again completed with the mandatory additional hydrogens to create a single alanine with a local geometry identical to that of an alanine in the helix. The same process creates tripeptides. A set of 2000 single (capped) alanine molecules and (capped) alanine tripeptides is created from the 2000 helix geometries. This data set should establish which minimum size of molecule must be trained for to accurately model much larger peptide chains.

### *Ab initio* treatment and machine learning of data sets 1, 2, and 3

The program GAUSSIAN09 calculated all wavefunctions at HF/6‐31 + G(d,p) level, which proved^55^ to be an excellent compromise between CPU time and accuracy. This fact is taken advantage of for the more than 22,000 wavefunctions calculated in this work, such that we do not have to invoke the B3LYP/apc‐1 level, which we have used in past publications. The diffuse functions on the heavy atoms occurring in the 6‐31 + G(d,p) basis set help in accurately portraying the electrostatics of biological systems. We note that hydrogen bonds do exist in the studied molecules and would ideally be included in a final atom‐typing project.

For Data Sets 2 and 3, the Cartesian coordinates are converted to a set of *3N‐6* non‐redundant descriptors of the geometry (termed “features”), where *N* is the number of atoms in the system. Features are then removed from the data set if they describe an atom outside of the desired fragment. For example, Figure 1 shows a fragment surrounding the alpha carbon (C_alpha_) of a central alanine residue, which contains only 10 of the 42 atoms in the tripeptide, thus reducing the data set from 3 × 42 − 6 = 120 features to 3 × 10 − 6 = 24 features. Not only does this reduction lead to faster training times but also means that the resulting model can be used for any other molecule containing this fragment, which includes any tripeptide with a central alanine. These fragments are described in full in a previous publication.^36^

Multipole moments are calculated for all atoms using the program AIMAll.^56^ The multipole moments give a complete description of the *ab initio* molecular electron density. The multipole moments are formulated in the non‐redundant spherical tensor formalism, up to the hexadecapole moment. Hence, there are 1 + 3 + 5 + 7 + 9 = 25 moments for each atom, making up *25N* values in total for a system with *N* atoms. All atomic multipole moments are rotated (using the method of Su and Coppens^57^) to their respective atomic local frames^32^ (ALFs) and all values in the database are normalized. An ALF is centred on an atom and the axes are oriented according to the position of that atom's highest priority bonded neighbors (according to Cahn‐Ingold‐Prelog rules). Thus, a given atom sees its environment independently of the molecule's rotation. The normalization process is not strictly required for kriging but is sensible when treating angles and distances on the same footing within a single model. The database is filtered for “undesirable” entries where geometries with large integration errors (L(Ω)) are filtered alongside those whose molecular net charge deviates significantly (>0.001 au) from the desired value (of zero for the neutral tripeptides used in this article). The remaining database entries (typically ∼1500 remain from an original set of 2000) are split into a “training set” and a “test set.” The tripeptides in this work each reserve 800 examples to training kriging models and the remaining examples are used for testing purposes.

### Machine learning

The machine learning method “kriging”^38^ is used to create models from the training data that can predict multipole moments using molecular geometries. Here we follow the method laid out by Jones et al.^58^ The kriging method is given fully in earlier works^59^ and so only its key concepts are covered here. The kriging method creates a relationship between a molecule's features (geometry) and output (multipole moments), $\hat{y}\left(x^{*}\right)$, which can be expressed as sum of the global term, $\hat{\mu }$, and a so‐called error (the summed term) as shown in eq. (1),

(1) $$\hat{y}\left(x^{*}\right)=\hat{\mu }+\sum_{i=1}^{n}a_{i}\cdot \varphi \left(x^{*}-x^{i}\right)$$

where $a_{i}$ is the *i*th element of $a=R^{-1}\left(y-1\hat{\mu }\right)$ where R is a matrix of error correlations between training points, and **1** is a column vector of ones. Thus, we take into account the correlation between the prediction example and all training examples, and assign importance to these correlations accordingly with φ. Indeed, if the prediction example is very close to a pre‐existing example in the training set, both examples are highly correlated and we can expect that they share a similar output value. In fact, the kriging predictor passes exactly through training points and a “perfect” prediction is achieved when attempting to predict the outputs for a known geometry. The prediction of any given point is greatly dependent on the training points that directly surround it in the feature space rather than those at increasing distances from the prediction point. If we cannot find a well‐correlated example in the training set, the output will tend toward the global term $\hat{\mu }$. This is a useful consequence of the kriging method when applied to chemical systems, giving sensible mean multipole moment values when a good prediction cannot be made. Some studies present kriging as a method for making good predictions on sparse data sets.^60^ Hence, it is not necessarily true that poorly correlated examples are unfit for use in modeling.

Finally, we can calculate the error between predicted multipole moments and their original (*ab initio*) counterparts. The error of a single atomic multipole moment is given in eq. (2),

(2) $$Q_{lm}^{\text{error}}=\left|Q_{lm}^{\text{orig}}-Q_{lm}^{\text{pred}}\right|$$

where *Q_lm_* is a moment of rank *l* and component *m*. The rank and component of a multipole moment indicates its shape and can be considered akin to typical atomic orbitals. A rank‐0 multipole moment can be considered analogous to an *s‐*orbital, rank‐1 moments to *p*‐orbitals, and so on. Each rank consists of 2*l* + 1 components (*m*) that take integer values between –*l* and +*l*. Thus *l* = 0 is a monopole and consists of a single moment whereas *l* = 1 is a dipole consisting of 3 moments (where *m* = −1, 0, and 1) and these are analogous to p‐orbitals: *p_x_, p_y_*, and *p_z_*. The focus of this work revolves around the atomic charge Q_00_ (*l* = m = 0) because it features most in multipolar electrostatics. Put more precisely, when going through all the possibilities of two multipole moments interacting with each other, the atomic charge (i.e., the zeroth moment) is combined with most other multipole moments (of higher rank). Second, given the long‐range nature of charge‐charge interactions they perpetuate over the longest distances, and hence, the largest number of interatomic interactions will still generate sizeable electrostatic (charge‐charge) energies. Moreover, the QTAIM charges have a chemical meaning^47^ unless they are interpreted naively, if not erroneously.^61^ The error involved with the reproduction of atomic charges is used to assess the quality of a kriging model and thus the success of exploited transferability.

## Results

### Question 1: on the grouping of amino acids

We begin by considering the question: *Can the natural amino acids be grouped according to their influence on a neighboring residue?* The answer to this question can mean the difference between needing to treat each of the 8000 possible tripeptide combinations as a unique case and treating the complete set of natural tripeptides as just a handful of different “groups” where each group is a collection of different tripeptides that have similar properties. Thus, a tripeptide “group” draws parallels to an atom's “type” in common force fields where an atom type is a collection of similar atoms that can share a single model or set of parameters.

Twenty tripeptides are constructed, as illustrated in Data Set 1 of Figure 2. All tripeptides (termed “AAX”) have identical geometries except for the third residue, which is substituted for each natural amino acid (the “X” in “AAX”). The effect on the central residue of each substitution can be measured as a comparison with the unsubstituted “AAA” tripeptide. Comparing each AAX tripeptide to AAA provides a basis for how we may group the amino acids when deciding which ones should share predictive models. Inspecting the atomic monopole moment (*Q*_00_) and the atomic kinetic energy *T*, we propose the following simple criteria for grouping the results:

- Δ*Q*_00_ < 0.005 au and Δ*T* < 10 kJ mol^−1^ then group A.
- Δ*Q*_00_ < 0.005 au and Δ*T* > 10 kJ mol^−1^ then group B.
- Δ*Q*_00_ > 0.005 au and Δ*T* < 10 kJ mol^−1^ then group C.
- Δ*Q*_00_ > 0.005 au and Δ*T* > 10 kJ mol^−1^ then group D.
- Proline is given a group of its own due to conventionally being considered a unique case because of its ability to invoke strong structural change to peptide secondary structure (group E).

Given the above criteria, neighboring residues can be assigned a group. We generated data for alanine's C_alpha_ *Q*_00_ with 20 different neighboring residues, now represented by four groups. Thus, we can make four kriging models for alanine's C_alpha_ *Q*_00_, one for each neighboring group. Compared to modeling a single amino acid, these new models contain information of the alanine's neighboring residues and should provide better predictions of alanine when part of a larger peptide chain. If the amino acids were not grouped, 20 highly specific models could be constructed, instead. However, we aim at striking a balance between accuracy, speed and ease of use in FFLUX.

The proposed grouping makes intuitive sense in most cases. It is not surprising that glycine, valine, alanine and isoleucine share a group (group A) because their sidechains are of similar composition. However, it is surprising that phenylalanine is also present in group *A* but leucine is not. That said, these data are based on comparison of single‐point (i.e., unoptimized) geometries and may not hold when the geometries are relaxed such that sidechains increase their ability to interact with neighboring sidechains. For a final, definitive atom‐typing, we suggest a more exhaustive data gathering and analysis of any present hydrogen bonds, which may also help to understand the grouping. A group consisting of glycine, alanine and valine makes intuitive sense and is backed up by our data, and so we proceed with this group (Data Set 2, Fig. 2) as a proof‐of‐concept.

### Question 2: on the influence of neighboring amino acids on kriging

Data Set 1 has proven that QTAIM properties of amino acids are sensitive to changes in neighboring residues and can be grouped accordingly. It is now that we can address our second question: *Can the influences of neighboring amino acids be accounted for using kriging machine learning?* To answer this, we must first establish that amino acids within a group share similar charges and that their responses to geometric change are similar. Second, it is important to build kriging models that can predict charges in the presence of amino acid neighbors of a particular group.

**Figure 3:**
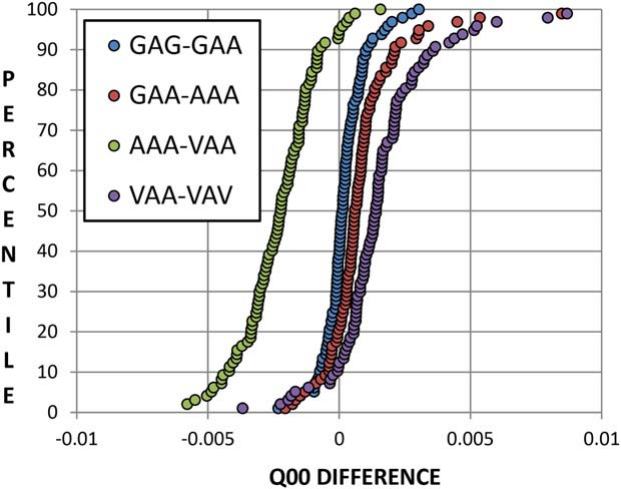
A set of 100 geometries for the tripeptide GAG undergoes a series of substitutions (GAG, GAA, AAA, VAA, VAV, see Data Set 2, Fig. 2) and its central alanine's C_alpha_ charges (*Q*_00_, given by the program AIMAll) are plotted as the change between each substitution. For example, the green series gives the difference in charges (obtained through *ab initio* calculation) between the tripeptides AAA and VAA in Data Set 2. [Color figure can be viewed at wileyonlinelibrary.com]

Data Set 2 has the added complexity of a residue being added to each side of the central alanine residue. It should be determined whether the side that the residue is added to has a profound effect, which must be taken into consideration by the model. The tripeptides in Data Set 2 are a progressive series where each tripeptide is compared to the next‐largest tripeptide. Residues are added in turn to either side of the central residue and the change this elicits in the central alanine residue is plotted in Figure 3.

**Figure 4:**
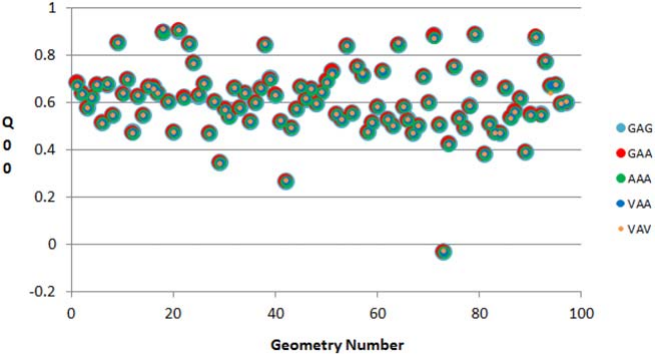
A set of 100 geometries for the tripeptide GAG undergoes a series of substitutions (GAG, GAA, AAA, VAA, VAV, see Data Set 2 in Fig. 2) and its central alanine's C_alpha_ charges (given by the program AIMAll) are plotted. Each geometry number has a single set of tripeptides (GAG, GAA, AAA, VAA, VAV) that share an identical backbone geometry. The difference in charge between geometries can be seen when comparing the results for different geometry numbers (*x*‐axis) while the difference in charge due to different terminal sidechains can be seen when comparing tripeptides of the same geometry number. [Color figure can be viewed at wileyonlinelibrary.com]

As the plots in Figure 3 deviate from zero, the step between the two tripeptides means a larger difference in the charge of the central C_alpha_. Series around zero difference such as GAG‐GAA and GAA‐AAA show that the corresponding tripeptides are extremely similar to one another (in terms of their effects on the central C_alpha_ charge). The change in going from VAA to AAA yields by far the largest change while VAV to VAA also deviates significantly from zero (although confusingly to the opposite direction). Changing a valine to an alanine involves eliminating two methyl groups compared to going from alanine to glycine (eliminating a single methyl group), thus a larger change is expected. When substituting glycine residues with alanines, it does not appear to be greatly significant which side of the central residue the substitution is made to, nor whether a single or double substitution is made. However, the change when substituting an alanine with a valine is comparatively large. Interestingly, a second alanine‐valine substitution appears to almost completely counteract the effect of the first alanine‐valine substitution. The positive aspect of this test is that, even at its most extreme, a difference in charge of only around 0.005 au is observed, although rare (∼0.1%) outliers do exist. These effects are not deemed significant enough to warrant different models for valine or models dependent on which side the substitution occurs. It is important to realize these changes in charge in the context of the charge's absolute value, shown in Figure 4.

As the series of tripeptides share the exact same set of local geometries, their properties can be directly mapped to one another, geometry‐by‐geometry. Thus, each geometry number has five different values for the charge on the central C_alpha_, one for each tripeptide in the series GAG, GAA, AAA, VAA, VAV, differing only in their terminal sidechains. It is evident that the charge of C_alpha_ depends much on molecular geometry, given the large range of charges seen with geometric change compared to changes in the terminal sidechains. Given that neighboring amino acids *G, A*, and *V* are similar in terms of their effect on the central residue, it is likely that multiple amino acid neighbors can be accounted for by the same model.

It remains to be seen whether a kriging model can predict the central C_alpha_ charges to an acceptable degree of accuracy. A kriging model will not reproduce charges for AAA perfectly, even when trained with AAA data. Making the jump to predicting a different tripeptide's charges will inevitably incur some additional error. To further complicate matters, a kriging model in FFLUX has *3N‐6* features where *N* is the number of atoms, meaning that two systems with a different number of atoms do not share the same number of features. Since the number of features in a kriging model is (at this time) static, a model cannot be used for multiple different systems without some modification. The solution is to make the number of features identical for each molecule removing features from each system until a common fragment is found as described earlier and depicted in Figure 1. Thus, we use fragments for all kriging models unless stated otherwise.

**Figure 5:**
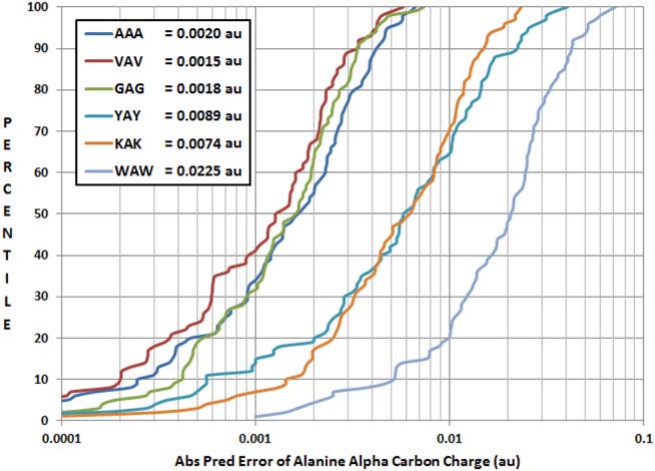
Comparison of kriging prediction of a central C_alpha_ atom's charge for six tripeptides. Tripeptides AAA, VAV, GAG are part of “group A” (Table 1). Tripeptides YAY, KAK, WAW are part of group D. The kriging model was trained for the tripeptide AAA (trialanine) using geometries that are not present in the test set. Mean prediction errors for the C_alpha_ charges of each tripeptide are given in au. [Color figure can be viewed at wileyonlinelibrary.com]

In Figure 5, a kriging model for predicting the charge of the central alanine C_α_ in a tripeptide is created using trialanine (AAA) data. This model can be used to predict the charge of alanine C_α_ atoms in other tripeptides and the prediction errors give a sense of the transferability of this model. As expected, the kriging machine learning is able to predict charges for the GAG or VAV molecule even when only trained using data for AAA, as both glycine and valine are of the same amino acid group (i.e., A) as alanine. In fact, predictions for GAG and VAV are surprisingly better than those for AAA, the system for which our model was created. When constructing kriging models of fragments, much of the specificity is lost due to lack of description of anything beyond an atom's local geometry and so it is conceivable that we obtain lower errors for systems other than the one specifically trained for.

**Table 1:** Charges (au) and kinetic energies (kJ mol^−1^) of the central C_alpha_ in the 20 tripeptides AAX (Data Set 1, Fig. 2), where *X* is a substituted residue.

AAX Tripeptide	*Q*_00_	Δ*Q*_00_	*T*	Δ*T*	Group
Alanine (A)	0.5506	0	98288.00	0	A
Cysteine (C)	0.5481	0.0024	98302.46	14.46	B
Aspartate (D)	0.5604	0.0098	98309.59	21.59	D
Glutamate (E)	0.5461	0.0045	98306.89	18.89	B
Phenylalanine (F)	0.5556	0.0049	98292.99	4.99	A
Glycine (G)	0.5562	0.0048	98295.42	7.41	A
Histidine (H)	0.5609	0.0103	98303.85	15.85	D
Isoleucine (I)	0.5546	0.0040	98296.09	8.09	A
Lysine (K)	0.5416	0.0090	98299.67	11.66	D
Leucine (L)	0.5456	0.0049	98301.49	13.49	B
Methionine (M)	0.5491	0.0015	98320.71	32.71	B
Asparagine (N)	0.5570	0.0064	98285.68	2.32	C
Proline (P)	0.5450	0.0055	98332.57	44.57	E
Glutamine (Q)	0.5439	0.0067	98293.09	5.08	C
Arginine (R)	0.5429	0.0077	98293.44	5.44	C
Serine (S)	0.5479	0.0027	98312.53	24.53	B
Threonine (T)	0.5500	0.0006	98311.72	23.71	B
Valine (V)	0.5525	0.0019	98297.51	9.51	A
Tryptophan (W)	0.5650	0.0145	98300.29	12.29	D
Tyrosine (Y)	0.5429	0.0077	98312.98	24.98	D

The geometry for each tripeptide is identical with exception to the substituted residue's sidechain. AAA is used as a reference molecule to which the other tripeptides are compared. A breakdown of the charges for different atoms in each tripeptide is given in Figure S1 in Supporting Information.

Meanwhile, predicting a tripeptide that contains amino acids outside of group *A*, results in much higher prediction errors, that is, 4 times higher for tyrosine (YAY) and lysine (KAK), and 11 times higher for tryptophan (WAW), all part of group *D*. Creating a model using data from all 6 tripeptides (GAG, AAA, VAV, YAY, KAK, WAW) lowers group *D* errors slightly but raises group *A* errors significantly (Figure S2 in Supporting Information). It is, of course, at the discretion of the force field user as to how much error is acceptable for their application of the force field, and this will affect how general their grouping of amino acids can be. We conclude that smart grouping of amino acids based on their influences as neighboring residues can lead to transferable models that accurately predict atomic charges.

### Question 3: on minimum peptide size to train kriging models of polypeptides

In an ideal world, a single amino acid could be representative of an amino acid in a peptide chain. Unfortunately, the problem remains that long peptide chains are expensive to calculate *ab initio* data for as well as being expensive to train models for, given their large number of features. It is now pertinent to ask the third question of this article: *What is the minimum size of a peptide that can be used to train kriging models for predicting atomic charges in a polypeptide chain?*

**Figure 6:**
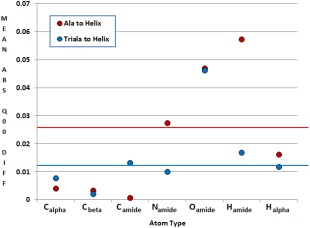
The difference in atomic charge between atoms in alanine, trialanine and a deca‐alanine helix. A solid line shows the overall mean difference in charges for each series (0.026 au and 0.012 au for alanine and trialanine, respectively). The mean difference across all 2000 geometries is expressed per atom. [Color figure can be viewed at wileyonlinelibrary.com]

To better understand how small a fragment can be while still being representative of a larger chain, Figure 6 compares the *ab initio* QTAIM charges of a deca‐alanine (3_10_) helix to those in smaller alanine units (single alanine and trialanine), where the mean difference across all 2000 geometries is expressed per atom.

As expected, it is clear from Figure 6 that trialanine has more common ground with deca‐alanine than a single alanine does (with differences of 0.012 au and 0.026 au, respectively) but this rule is not as universal as expected. It is important to note that deca‐alanine has important features that are not represented by the single or trialanine molecules such as the hydrogen bonding interaction that occurs between distant residues of the helix, and these interactions are likely to have profound effects on an alanine atom's multipole moments. Additional features may be added to the kriging models in future to account for such important additional interactions.

**Figure 7:**
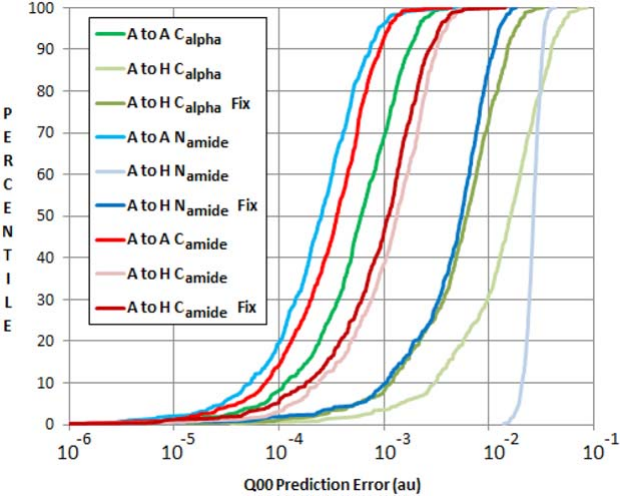
Kriging models for the C_alpha_ of a single alanine predicting C_alpha_ charges in alanine (A) and in a deca‐alanine helix (H). “μ Fix” predictions show predictions of helix C_alpha_ properties with altered μ values in the kriging models. [Color figure can be viewed at wileyonlinelibrary.com]

Given that the single alanine is not as far removed from the helix as expected, it may be possible to train alanine kriging models to provide sufficiently accurate predictions for atoms in deca‐alanine, as shown in Figure 7 for a selection of atoms.

The “A to A” predictions use kriging models built using alanine data to predict alanine data. These three models predict with low error (to the left of the S‐curve). However, the three “A to H” predictions (alanine models predicting helix charges) create much higher prediction errors. Despite having similar mean charges across the set of geometries, alanine C_alpha_ models do not appear to be innately suited to predicting helix properties. Fortunately, an alanine model can be further tailored toward deca‐alanine predictions by altering its mean background prediction value. A kriging model maps the output (charge) for a particular geometry as an “error” from a mean background value (termed μ) based on the charges of all trained geometries. Thus, if μ takes a different value for a single alanine than it does for a deca‐alanine, all predictions will be wrong by that difference. We can, in theory, remedy this by changing μ (a single value) in the kriging model to convert an alanine model to a “fixed μ” alanine model, which emulates a helix model as seen by the “fix” plots seen in Figure 7.

The “fixed” models show almost‐universally lower errors. In the most dramatic example, the kriging prediction error for N_amide_ (blue curves, Fig. 7) is lowered by an order of magnitude. At its worst, the μ fix does not appear to have a significant effect. Note that for such a fix to be possible, one must have some knowledge of what μ should be for their predicted system, and we achieved this by already having created models trained on deca‐alanine data. Without data for deca‐alanine, correcting the μ value could well be a case of trial and error, or perhaps requiring a machine learning model of its own. Knowing μ means knowing the mean value for the multipole moment to be predicted and thus a database of these values would need to be constructed for long‐term use.

**Figure 8:**
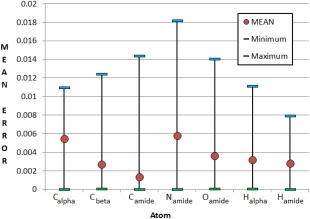
Prediction of charges in a deca‐alanine helix from models trained using trialanine data (AAA to H). All charges are expressed in atomic units. [Color figure can be viewed at wileyonlinelibrary.com]

It is deduced from Figures 6 and 7 that trialanine is a better base for building kriging models with the intent of predicting atom properties in the deca‐alanine helix. Not only do trialanine's charges lie generally closer to those found in deca‐alanine, but trialanine models can also capture effects from neighboring residues. The minimum, maximum and mean prediction errors for the atomic charges in deca‐alanine, when predicted by trialanine models, are given in Figure 8.

Each atom has a mean prediction error below 0.006 au and an error below 1% of the charge range (Figure S3 in Supporting Information) that the respective atoms span throughout the training set. The trialanine models predicting helix charges yield 0.006 au, 0.006 au, and 0.002 au for atoms C_alpha_, N_amide_, and *C*_amide_, respectively, which is a marked improvement over the corresponding single‐alanine models in Figure 7 (0.021 au, 0.027 au, 0.002 au mean error for the same atoms). For many atoms, maximum errors are far from the mean compared to minimum errors and are comparatively few in number. This same trend can be seen in Figure 7 where S‐curves have a sharp tail at the top‐right of each curve, indicating very few examples with unusually high prediction error. Each set of predictions is still of higher error (approximately double) than if a kriging model had been trained using deca‐alanine helix data.

It should be noted that the time‐saving effect of transferable kriging models is a two‐fold effect. As with all force fields, transferability is devised to generalize the force field and allow predictions of new systems *without reparameterization or without taking parameters from a system that is unfeasibly large with respect to ab initio calculation*. Meanwhile, the transferable kriging models also have far fewer features than regular models, which drastically lowers the dimensionality of the training problem and thus speeds up the training process. Table 2 lists the number of Particle Swarm Optimisation (PSO) iterations^29^ of the kriging training, and the training times (in seconds) for creating kriging models trained with “fragment sets” (training sets with reduced features based on a fragment of a larger molecule such as that in Fig. 1) and with “regular sets.”

**Table 2:** Computation times (in seconds) for the kriging model training of various atomic charges.

	C_alpha_	C_beta_	C_amide_	N_amide_	O_amide_	H_alpha_	H_amide_
**Fragment**							
Regular	15514	4719	13287	5277	7004	3878	10969
Iterations	4355	1491	3806	1556	1093	1203	3119
**Full**							
Transfer	906	763	851	514	992	349	438
Iterations	623	528	608	389	792	278	283
% Time	5.84	16.17	6.40	9.74	14.16	8.99	3.99

The “FULL” section provides data for regular training sets while the “FRAGMENT” section provides data for reduced training sets.

The regular training sets have 120 features whereas transferable sets have only 24, thus reducing the number of features by 5 times and results in a reduction of training time by approximately a factor of 10. Training times appear to become more predictable as the number of features is smaller, which is perhaps due to a simpler (and perhaps smoother) feature space that makes model optimization easier. Indeed, the number of iterations the PSO requires to converge is consistently smaller for the transferable training sets. In many cases, the training process for an entire molecule can only be considered to be as fast as the slowest training of its individual atomic models and so it is more useful to consider the longest training time rather than a mean average of the times. Although the simplification of the kriging model by reducing its number of features does carry an error penalty in charge prediction, the large time saving and ability to build transferable models makes this a justifiable sacrifice.

## Conclusion

We have set out to test three related concepts, which we briefly recap.

First, the residue neighboring an amino acid is significant to the amino acid's atomic charges. Amino acids can be grouped according to their influence as a neighbor and we have suggested five groups, based on the QTAIM properties for atomic charge and kinetic energy, for the 20 natural amino acids.

Second, a kriging model is capable of predicting amino acid properties in a peptide chain as long as the neighbors of a given amino acid belong to the same group. Thus, when training for the tripeptide AAX (where A is alanine and X is any natural amino acid), predicting outside one of residue X's proposed group yields a prediction error up to 11 times larger than that from a prediction within X's group.

Third, we have explored the minimum feasible system size of a training data set for the prediction of larger systems. Given single alanine and trialanine atomic charges, it was found that trialanine charges are most similar to deca‐alanine atomic charges, which is likely due to the presence of neighboring groups. Tripeptide‐trained models still return twice the error compared to models trained using deca‐alanine data but the computational cost is 5 to 20 times lower. Furthermore, kriging models were more successfully transferred to deca‐alanine when the μ value (indicating the “background” charge that a kriging model expects) was altered to fit mean deca‐alanine charges. Deca‐alanine charges, even without altering μ, can be predicted from trialanine data with a mean error of around 0.004 au (an accuracy of > 99%).

In conclusion, the consideration of neighboring residues is valuable for the accurate portrayal of atomic charges in large peptide systems. Even a crude inclusion of these neighbor effects into atom‐typing can give much better prediction accuracy. Meanwhile, although a small fragment is capable of describing a charge in a long peptide chain, that fragment should be trained using tripeptide data rather than single amino acid data. This is because, generally, tripeptide models give better prediction accuracy and can include neighboring residues to increase specificity of the model.

By combining the conventional wisdom of atom‐typing and transferability with modern electrostatics and machine learning, FFLUX represents the groundwork for future improvements in applying force fields to large biological systems. We reach toward a future of force field development grounded in accurate, directional electrostatics that are transferable and well understood.

## Supporting information

Supporting Information
