# Supplementary material for: Toward amino acid typing for proteins in FFLUX
Source: J Comput Chem. 2016 Dec 19;38(6):336–45. doi: 10.1002/jcc.24686 (PMC6681421; doi:10.1002/jcc.24686)
Supplement: Supplementary file 1 — Supporting Information [file JCC-38-336-s001.docx]

**Supporting Information**

**Towards amino acid typing for proteins in FFLUX**

**Timothy L. Fletcher and Paul L.A. Popelier**

**
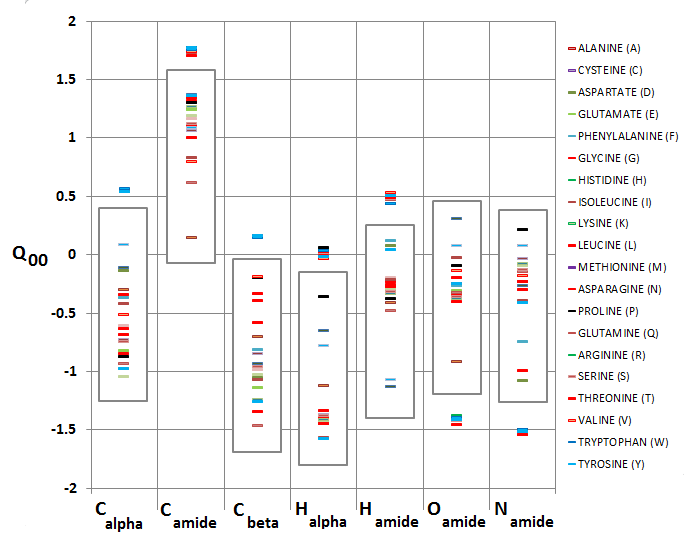
**

**Figure S1.** The net atomic charge Q_00_ of atoms in the central alanine amino acid with different neighbouring residues. The figure is magnified inside the boxes to show details of the closely-packed data. Charged amino acids are in various shades of green, aromatic amino acids are in shades of blue, neutral amino acids are in shades of red, and proline is black. Compared to the absolute value of a charge, the effects of a neighbouring residue are minor. However, there is enough fluctuation in all atoms that the neighbouring residue would be an important consideration for accurate modelling and especially for mechanical insight. Atoms involved in amide groups exhibit a much wider range of changes than atoms at the core of the residue. We hypothesize that the closer an atom is to the changing neighbour, the more influence it feels. However, hydrogen atoms (both H_alpha_ and H_amide_) suffer the greatest perturbations, perhaps due to through-space effects from neighbouring sidechains.


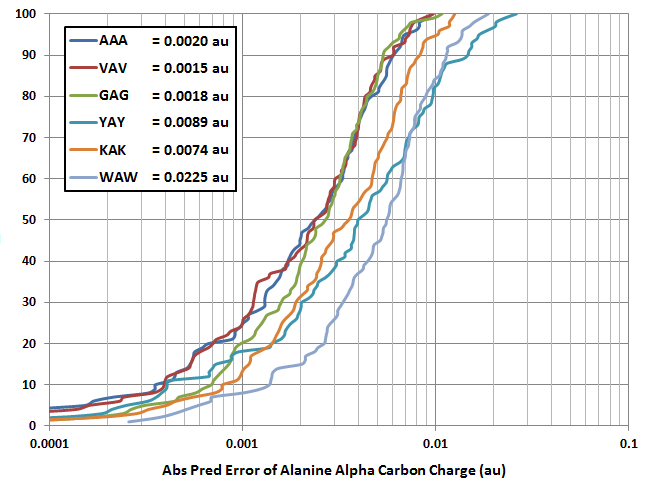


**Figure S2.** Q_00_ on the C_alpha_ of the central alanine of different tripeptides is predicted using a model that is built using data for AAA, VAV, GAG, YAY, KAK, WAW. As expected, the model is less specific than one trained just using group A tripeptides (AAA, GAG, VAV), giving higher errors for those tripeptides. However, errors are lower for YAY KAK and WAW predictions as these are better represented in the training data. With only four groups of neighbouring residue (A,B,C,D) suggested in this study, we conclude it would be beneficial to make a model for each group for heightened predictive accuracy.


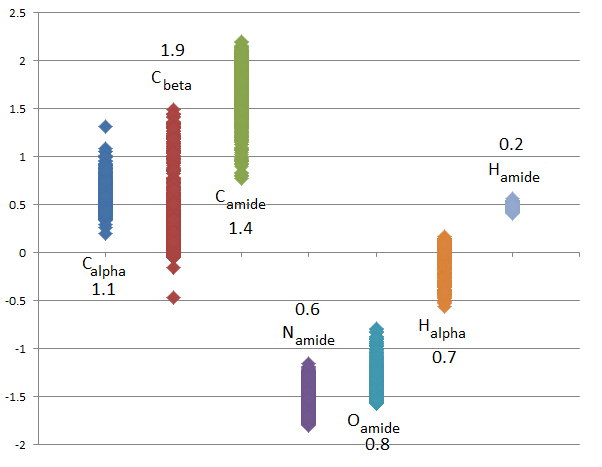


**Figure S3.** Charges of atoms in the trialanine training set used for predicting deca-alanine helix charges. The range of each set is given as a number beside that set’s label.
